# A Multi-Pathogen Screening of Captive Reindeer (*Rangifer tarandus*) in Germany Based on Serological and Molecular Assays

**DOI:** 10.3389/fvets.2019.00461

**Published:** 2019-12-20

**Authors:** Javier Sánchez Romano, Lisa Grund, Anna Obiegala, Ingebjørg H. Nymo, Francisco Javier Ancin-Murguzur, Hong Li, Nina Król, Martin Pfeffer, Morten Tryland

**Affiliations:** ^1^Arctic Infection Biology, Department of Arctic and Marine Biology, UiT The Arctic University of Norway, Tromsø, Norway; ^2^Zoo Duisburg AG, Duisburg, Germany; ^3^Institute of Animal Hygiene and Veterinary Public Health, Veterinary Faculty, University of Leipzig, Leipzig, Germany; ^4^The Norwegian Veterinary Institute, Tromsø, Norway; ^5^Northern Populations and Ecosystems, Department of Arctic and Marine Biology, UiT The Arctic University of Norway, Tromsø, Norway; ^6^Animal Disease Research Unit, USDA-Agricultural Research Service and Department of Veterinary Microbiology and Pathology, College of Veterinary Medicine, Pullman, WA, United States

**Keywords:** herpesvirus, pestivirus, *Schmallenberg virus*, bluetongue virus, *Brucella*, *Toxoplasma gondii*, *Neospora caninum*, *Anaplasma phagocytophilum*

## Abstract

Captive reindeer in German zoos and wildlife parks live outside their natural geographic range and are exposed to a variety of viral, bacterial and protozoan pathogens, some host-specific and some which they are not exposed to in their native habitat. Reindeer blood samples and ticks collected in 2013 from 123 reindeer at 16 different zoological facilities were available from a previous study. The aims of this study were to assess the serological status of these animals with regards to various microorganisms as well as to test ticks (*Ixodes ricinus*) and blood samples for the presence of *Anaplasma* spp. DNA in order to evaluate the exposure of captive reindeer in Germany to a variety of pathogens. A total of 119 or 118 serum samples were screened (ELISA) and antibodies were detected (seropositive/tested, prevalence, confidence interval) against alphaherpesvirus (24/119, 20.3%, CI: 13.9–28.3), bluetongue virus (BTV; 4/119, 3.4%, CI: 1.0–8.7), malignant catarrhal fever related gammaherpesvirus (MCFV-related gammaherpesvirus; 7/119, 5.9%, CI: 2.7–11.9), pestivirus (5/118, 4.2%, CI: 1.6–9.8), Schmallenberg virus (SBV; 70/118, 59.3%, CI: 50.3–67.8), smooth *Brucella* spp. (1/118; 0.9%, CI: 0–5.1), *Neospora caninum* (5/118, 4.2%, CI: 1.6–9.8), and *Toxoplasma gondii* (62/119, 52.1%, CI: 43.2–60.9). These results suggested the exposure of reindeer to all tested pathogens. Moreover, real-time PCR for *Anaplasma phagocytophilum* targeting the partial *msp2* gene was performed on DNA extracted from whole blood samples from reindeer (*n* = 123) and from ticks (*n* = 49) collected from 22 reindeer in seven different facilities. In addition to the real-time PCR, a semi-nested PCR for the partial *groEL* gene, and a nested PCR targeting the partial 16S rRNA gene were performed. DNA of *A. phagocytophilum* was detected in 17 reindeer (13.8%) and 15 ticks (30.6%). Three of the five reindeer with ticks having *A. phagocytophilum* DNA also had such DNA in blood. These results indicate that captive reindeer can be exposed to several ruminant pathogens that they hitherto had no known exposure to through their natural geographical distribution and habitats as shown for *Culicoides*-borne BTV and SBV. Further, captive reindeer may serve as reservoir hosts for pathogens circulating in local domestic, captive, and wild ruminant species and populations and arthropod vectors.

## Introduction

Reindeer and caribou (*Rangifer tarandus*), often called *Rangifer*, are wild or semi-domesticated ruminants of the family *Cervidae*, with seven different subspecies distributed in arctic and subarctic ecosystems (1). Reindeer and caribou are also displayed in zoological and wildlife parks all around the globe (2). Depending on migration routes, ecosystems and also herding conditions, *Rangifer* are exposed to numerous infectious agents, of which some may cause disease or may be of zoonotic concern (3). Assessing health indicators and diseases in wild ungulate populations is challenging and often neglected, and it is not fully known which established and potential pathogens are circulating (4–6). In addition to *Rangifer* host-specific pathogens, ruminant species kept in zoos and parks may be exposed to a broad variety of emerging and zoonotic pathogens to which they are not exposed in their native habitat, reflecting the close contact with other host species and locally occurring arthropod vectors.

Cervid herpesvirus 2 (CvHV2) is an alphaherpesvirus that is enzootic in the semi-domesticated reindeer populations in Fennoscandia and in caribou in North America (7, 8). In reindeer, CvHV2 can cause infectious keratoconjunctivitis (IKC) (9) as well as respiratory disease (10).

Bluetongue virus (BTV; genus *Orbivirus*, family *Reoviridae*) may cause bluetongue (BT) in domestic and wild ruminants (11, 12). BTV is transmitted by biting midges of the genus *Culicoides*, but can also be transmitted via direct contact or transplacental transmission (11, 13).

Malignant catarrhal fever viruses (MCFV) is a group of 10 different herpesviruses classified in the genus *Macavirus*, subfamily *Gammaherpesvirinae* (14). Domestic sheep (*Ovis aries*) are considered to be reservoir hosts of ovine herpesvirus 2 (OvHV2), while goats (*Capra aegagrus hircus*) are hosts of caprine herpesvirus 2 (CpHV2) (14). MCFV may lead to fatal disease in several ruminant species including reindeer (15, 16). Anti-MCFV-related gammaherpesvirus antibodies have been detected in semi-domesticated reindeer in Fennoscandia, with a prevalence of 3.5–3.8% (17, 18).

Pestivirus A (formerly Bovine Viral Diarrhea Virus 1; BVDV1) and D (formerly Border Disease Virus; BDV) are members of the genus *Pestivirus*, family *Flaviviridae* (19). The susceptibility of reindeer to pestivirus A infection has been experimentally demonstrated (20) and a pestivirus (V60-Krefeld), phylogenetically and antigenically closely related to pestivirus D and reclassified as a genotype of this species (19), was isolated from a captive reindeer in Duisburg Zoo, Germany (21, 22). Serological screenings have demonstrated that pestivirus infections are present in semi-domesticated reindeer in Fennoscandia (23, 24) and in caribou in North America (25).

Schmallenberg virus (SBV; genus *Orthobunyavirus*, family *Peribunyaviridae*) was isolated for the first time from a cow in the city of Schmallenberg, Germany, in 2011, and spread rapidly to most of Europe (26, 27). The virus is transmitted by arthropod vectors, of which biting midges (*Culicoides* spp.) are considered the most important (28). In domestic ruminants, the virus may cause mummified fetuses, premature birth, and congenital malformations (27–29). No reports exist on SBV in reindeer, or in any other host species in regions inhabited by wild or semi-domesticated reindeer (30).

The genus *Brucella* (family *Brucellaceae*) consists of multiple species with a wide variety of host preferences (31). *Brucella suis* biovar 4 is the causative agent of brucellosis in *Rangifer* (32) and may cause clinical signs most often associated with the reproductive systems (abortion, stillbirth, male sterility) and joints (synovitis and bursitis). The disease is also known as “*rangiferine*” brucellosis and continues to be an important public health concern in the Arctic, where many people depend on reindeer and caribou for their subsistence (33).

*Toxoplasma gondii* and *Neospora caninum* are both obligate intracellular coccidia with carnivore definitive hosts and a variety of intermediate hosts, including wild and domestic ruminants (34, 35). Several serological screenings have been performed in wild and captive cervids, with a seroprevalence varying from 0.9 to 34.0% for *T. gondii* and from 0.5 to 40.5% for *N. caninum* (34–38).

*Anaplasma phagocytophilum* is a tick-borne obligate intracellular bacterium that causes granulocytic anaplasmosis in humans but also in mammals such as dogs and ruminants (39). Wild ruminants (genus *Cervus, Capreolus* and *Rupicapra*) are expected to be main reservoirs in Europe (40). There is only one report of *A. phagocytophilum* infection in reindeer from Norway (41) but a high prevalence (80.0%) of *Anaplasma ovis* was found by PCR in reindeer from Mongolia (42). An experimental infection of *Rangifer t. tarandus* with *A. phagocytophilum* resulted in severe clinical symptoms such as anemia and inappetence and one fatal case (43).

The aim of this study was to investigate the possible circulation of all tested pathogens in the selected captive reindeer populations in Germany, as well as the possible role of captive reindeer as reservoir hosts for important pathogens of other domestic, captive and wild ruminant species.

## Materials and Methods

### Animals and Sampling

**Figure 1:**
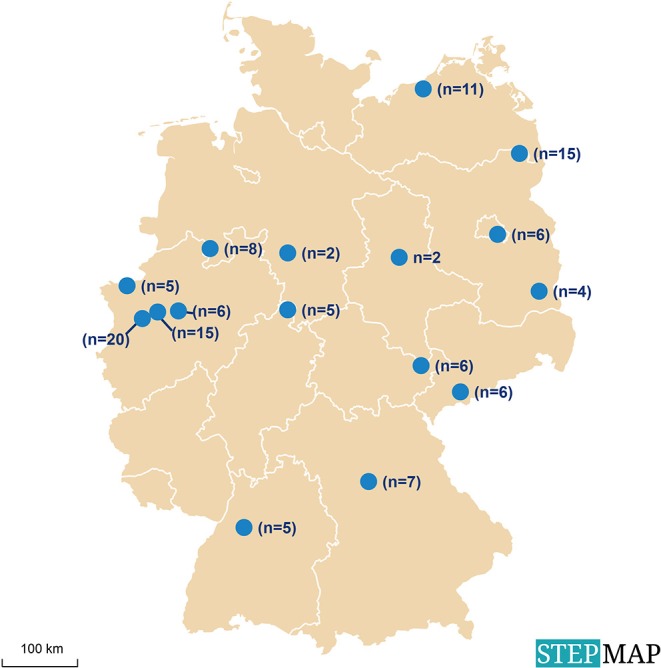
Locations of animal facilities with reindeer (*Rangifer t. tarandus*) in Germany selected for sampling for the investigation of exposure to infectious agents.

In a previous study on *Babesia* spp. in reindeer (*R. t. tarandus*) in zoos and wildlife parks in Germany (44), 33 facilities with reindeer were identified and contacted. Sixteen of these facilities, distributed throughout the country and holding ~50% of all captive reindeer in Germany at that time, were chosen as sampling sites (Figure 1). Samples were taken from 123 reindeer of different age and sex (Table 1). None of the animals showed any clinical signs of disease at the time of sampling. Husbandry characteristics and individual medical histories were obtained for each animal via standardized questionnaires and registered in a database.

**Table 1:** List of sampling sites of captive reindeer (*Rangifer t. tarandus*) with number and age of animals tested.

**Town**	**Age**			**Total**
	**<1 year**	**1–2 years**	**>2 years**	
Isselburg	2	0	3	5
Berlin	0	1	5	6
Cottbus	0	1	3	4
Dortmund	0	1	5	6
Duisburg	6	8	6	20
Gelsenkirchen	5	1	9	15
Gera	2	2	2	6
Magdeburg	0	0	2	2
Nürnberg	0	2	5	7
Osnabrück	4	0	4	8
Pforzheim	1	0	4	5
Rostock	4	0	7	11
Sababurg	1	1	3	5
Schenkenberg	3	2	10	15
Springe	0	1	1	2
Waschleithe	0	5	1	6
Total	28	25	70	123

*Names correspond to Figure 1 (map)*.

### Blood and Tick Sample Collection

EDTA blood samples and serum samples from reindeer were available from a previous study (44). For the same previous study, 49 ticks were collected from 22 reindeer in seven different facilities as previously described (44). All ticks were identified as adult stages of *Ixodes ricinus* (14 males and 35 females). Whole blood samples, serum samples and ticks were stored at −20°C until further examination (44). For our study, 118 samples could be tested against the complete panel, while one of the samples was tested against four pathogens only, due to volume restrictions.

### DNA Extraction

DNA was extracted from whole blood samples and ticks as previously described (44) and DNA concentration was measured by spectrophotometry (Nanodrop 2000c, Thermo Scientific, USA).

### Serology

Sera were tested by ELISA for the presence of antibodies against seven pathogens known to cause disease in reindeer or other cervids (Table 2).

**Table 2:** Enzyme-linked immunosorbent assays (ELISA) used for investigating captive reindeer (*Rangifer t. tarandus*) in Germany for exposure to infectious agents.

**Detected antibodies**	**Method**	**Dilution**	**Validated for reindeer**	**Reference**	**Variables analyzed by MCA^a^**
Alphaherpesvirus	BoHV1 blocking ELISA kit (LSI, Lissieu, France)	1:2	Yes	(45)	1, 2, 3, 4
Bluetongue virus (BTV)	ID Screen® Bluetongue virus Competition ELISA (IDVet, Montpellier, France)	1:2	No		1, 2, 3, 5
*Brucella* spp.	Protein A/G indirect enzyme-linked Immunosorbent assay for the detection of anti-*Brucella* antibodies	1:50	Yes	(46)	1, 2, 3, 4
MCFV-related gammaherpesvirus	Direct competition ELISA for the detection of antibodies against the MCFV group	1:5	Yes	(47)	1, 2, 3, 4
*Neospora caninum*	Indirect multi-species ELISA kit for the detection of anti-*Neospora caninum* antibodies (IDVet, Montpellier, France)	1:10	No		1, 2, 4
Pestivirus	SERELISA™ BVD p80 Ab Mono blocking (Synbiotics Europe SAS, Lyon, France)	1:10	Yes	(48)	1, 2, 3, 4
*Schmallenberg virus* (SBV)	ID Screen® Schmallenberg virus Competition multi-species ELISA (IDVet, Montpellier, France)	1:1	No		1, 2, 3, 5
*Toxoplasma gondii*	ID Screen® Toxoplasmosis Indirect multi-species ELISA (IDVet, Montpellier, France)	1:10	No		1, 2, 3, 4, 5, 6, 7

a *Variables analyzed in the multiple correspondence analysis (MCA) were selected considering the characteristics of the different pathogen. Selected variables included sex (1; male, female), age class (2; calf, juvenile, adult), origin (3; born at the facility, born in Germany, born abroad), neighbor species (4; cervids, other artiodactyls, perissodactyls, carnivores and birds), vegetation (5; yes, no), feed stock nearby (6; yes, no), rodent control (7; yes, no), anti-parasitic treatment (8; yes, no). Full database is available in doi: 10.18710/4PQKKQ*.

### Molecular Testing

The DNA concentration of the extracts from blood and tick samples were adjusted to 25 ng/μl for each sample. Diluted DNA samples were analyzed for the presence of DNA specific to *A. phagocytophilum* by PCR. Samples were tested with a real-time PCR protocol for the partial *msp2* gene (77 bp) (49). Samples yielding a positive result were further analyzed by semi-nested PCR for the partial *groEL* gene (573 bp) (50) and by a nested PCR targeting the partial 16S *rRNA* gene (546 bp) (51). PCR products were purified (QIAquick PCR Purification Kit; Qiagen, Hilden, Germany) prior to sequencing as previously described (40). Sequences were analyzed, aligned and compared with sequences deposited in GenBank with BLASTn (National Center for Biotechnology Information, Bethesda MD, USA) using the Bionumerics Software (Version 7.6.1. Applied Maths, Inc., Austin, TX, USA).

### Statistical Analysis and Multivariate Correspondence Analysis

Confidence intervals (95% CI) for seroprevalence rates were determined by the Clopper and Pearson method with Graph Pad Software (Graph Pad Software Inc., San Diego, Ca., USA). Independence of compared samples was analyzed with the chi-squared test.

Multiple correspondence analysis (MCA) (52) was used to explore relationships between the explanatory variables using the package *FactoMineR* (53) in R Core Team (54). Individual MCA analyses were performed on each pathogen using a combination of the following parameters: sex, age class (calf <1 year old, juvenile between 1 and 2 years old, adult >2 years old), origin of the individual (i.e., imported from abroad, translocated from other parts of Germany or in-house zoo-bred), neighboring species (grouped in cervids, other artiodactyla, perissodactyla, carnivora and birds), presence of vegetation, rodent control and antiparasitic treatment (Ivermectin) (Table 2).

## Results

### Serology

Due to small volumes of sera available, not all samples from the 123 individual reindeer were available for testing in all assays. Results are summarized in Table 3.

**Table 3:** Presence and prevalence of antibodies against a range of infectious agents in captive reindeer (*Rangifer t. tarandus*) in Germany (2013), presented as seropositive/tested (percentage and confidence interval) by age group.

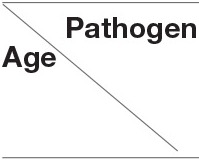	**Alphaherpesvirus^a^**	***Brucella* spp**.	**Bluetongue virus**	**MCFV-related gammaherpesvirus**	***Neospora caninum***	**Pestivirus**	**Schmallenberg virus^a^**	***Toxoplasma gondii*^a^**
Calves (<1 year)	3/28 (10.7%; 2.9–28.0)	0/28 (0%; 0–14.3)	0/28 (0%; 0–14.3)	2/28 (7.1%; 0.9–23.7)	0/28 (0%; 0–14.3)	1/28^c^	4/28 (14.3%; 5.1–32.1)	2/28 (7.1%; 0.9–23.7)
Juvenile (1–2 years)	3/24 (12.5%; 3.5–31.8)	0/24 (0%; 0–16.3)	0/24 (0%; 0–16.3)	0/24 (0%; 0–16.3)	0/24 (0%; 0–16.3)	1/24 (4.2 %; 0–21.9)	14/24 (58.3%; 38.8–75.6)	8/24^b^ (33.3%; 17.8–53.4)
Adult (>2 years)	18/67 (26.9%; 17.7–38.6)	1/66 (1.5%; 0–8.9)	4/67 (6.0%; 1.9–14.8)	5/67 (7.5%; 2.9–16.7)	5/66 (7.6%; 2.9–16.9)	3/66 (4.6%; 1.0–13.1)	50/66 (75.8%; CI 64.1–84.6)	51/67 (76.1%; 64.6–84.8)
Total	24/119 (20.2%; CI 13.9–28.3)	1/118 (0.9%; CI 0–5.1)	4/119 (3.4%; CI 1–8.7)	7/119 (5.9%; CI 2.7–11.9)	5/118 (4.2%; CI 1.6–9.8)	5/118 (4.2%; CI 1.6–9.8)	70/118 (59.3%; CI 50.3–67.8)	62/119 (52.1%; CI 43.2–60.9)

*Results are displayed as number of positive individuals/number of individuals tested (%; 95% CI = Confidence interval of 95%)*.

a *Seroprevalence was significantly higher for pathogens of which the prevalence was positively linked with the age classes (χ^2^ = 225.5; p < 0.0001)*.

b *1 sample was classified as “doubtful,” retested with identical result and was considered negative*.

c *2 samples were classified as “doubtful,” retested with identical result and were considered negative*.

#### Alphaherpesvirus

24 samples were positive for the presence of antibodies against alphaherpesviruses (*n* = 119; 20.3%; CI: 13.9–28.3). Seroprevalence increased with age, with 10.7% (CI: 2.9–28.0) for calves, 12.5% (CI: 3.5–31.8) for juveniles and 26.9% (CI: 17.7–38.6) for adults.

#### BTV

Four out of 119 animals had antibodies against BTV (3.4%; CI: 1.0–8.7). All seropositive animals were adults (*n* = 67).

#### MCFV-Related Gammaherpesvirus

Antibodies against MCFV-related gammaherpesvirus were detected in seven out of 119 animals (5.9%; CI: 2.7–11.9).

#### Pestivirus

Five out of 118 serum samples were positive for the presence of antibodies against pestivirus (4.2%; CI: 1.6–9.8).

#### SBV

Antibodies against SBV were detected in 70 out of 118 reindeer (59.3%; CI: 50.3–67.8), with increasing prevalence with age; 14.3% (CI: 5.1–32.1) in calves, 58.3% (CI: 38.8–75.6) in juveniles and 75.8% (CI: 64.1–84.6) in older animals.

#### Brucella spp.

Anti-*Brucella* antibodies were detected in one healthy adult female reindeer (*n* = 118; 0.9%; CI: 0–5.1).

#### Neospora caninum

Antibodies against *N. caninum* were present in five of 118 reindeer (4.2%; CI: 1.6–9.8).

#### Toxoplasma gondii

62 out of 119 tested reindeer were positive for the presence of antibodies against *T. gondii* (52.1%; CI: 43.2–60.9). Seroprevalence increased with age, being 7.1% (CI: 0.9–23.7) in calves, 33.3% (CI: 17.8–53.4) in juveniles, and 76.1% (CI: 64.6–84.8) in adult reindeer.

The seroprevalence was significantly higher for pathogens of which the prevalence was positively linked with age (χ^2^ = 225.5; *p* < 0.0001), i.e., alphaherpesvirus, SBV and *T. gondii*.

### Molecular Testing on Anaplasma phagocytophilum

Seventeen of 123 reindeer (13.8%; CI: 8.7–21.1) were positive for *A. phagocytophilum* DNA by real-time PCR targeting the *msp2* gene. Analysis of the *groEL* gene yielded positive results for nine of these. PCR amplicon sequences from six out of these nine reindeer showed 99–100% identity to ecotype 2 (55), whereas the remaining three showed 99% similarity to ecotype 1. Thirteen of the 17 samples (76.4%) positive with the *msp2* gene also generated PCR amplicons when testing with primers targeting the *16s rRNA* gene. Six of these 13 samples showed 100% identity to strain “16S−22 Y” (51), four showed 99–100% identity to strain “16S−21 X,” two showed 99% similarity to “16S−8 J” and one showed 100% identity to strain “16S-20 W.”

Fifteen out of 49 ticks (30.6%; CI: 19.4–44.6) were PCR-positive for *A. phagocytophilum*. These 15 ticks had been collected from five individual reindeer from two different facilities. The prevalence for *A. phagocytophilum* did not differ significantly (*P* = 0.7349) between males (35.7%) and females (28.6%). Due to the high CT value obtained from ticks, only one tick yielded a positive result concerning the *16S rRNA* gene (“16S-21 X”), matching the result of the reindeer from which it was collected. Three of the five reindeer with ticks having *A. phagocytophilum* DNA also had such DNA in the blood.

### Multivariate Analysis

*Brucella*, pestivirus, MCFV-related gammaherpesvirus, BTV and *Neospora* antibodies were found in few individuals only, thus the interpretation of MCA was not conclusive due to the scarcity of data. MCA showed an association between having alphaherpesvirus antibodies and being imported to Germany from abroad. *Schmallenberg virus* antibodies were present predominantly in adult individuals, thus age was identified as the most relevant variable, followed by the presence of vegetation in the enclosure. *Toxoplasma*, on the other hand, was positively related to presence of neighboring cervids and vegetation, and negatively related to the presence of carnivores in neighboring enclosures. Finally, the detection of *Anaplasma*-DNA was positively associated with being corralled with other herbivores. Detailed MCA results are displayed in Appendix 1 (Supplementary Material).

## Discussion

The reindeer alphaherpesvirus (CvHV2) is widespread among wild and semi-domesticated reindeer populations (7, 8). The lower seroprevalence against alphaherpesviruses in adult reindeer reported in this study (26.9%) as compared with seroprevalence found in adult wild and semi-domesticated reindeer (~50.0%) (3, 7), may suggest that captive animals are less prone to stress events that could facilitate reactivation and spread of a latent herpesvirus infection. However, if only the facilities in which there are seropositive animals are selected, seroprevalence in adult animals increases to 50.0% (*n* = 36), ranging between 22.2 and 100.0%, and MCA analysis pointed to the importance of importing reindeer from abroad in the transmission of alphaherpesviruses. This finding suggests that facilities with CvHV2-seronegative reindeer have most probably avoided the contact of their reindeer with CvHV2-infected animals, either through the import of unexposed animals or by replenishing their stock through their own breeding program.

BTV can infect a wide range of domestic animals, but also most species of wild ruminants are susceptible (11, 12, 56). Bluetongue epizootics occurred in Germany in 2006–2009, with more than 1.8 million domestic ruminants exposed to the virus and generating high mortality rates among infected sheep (57). Despite the appearance of the disease in Scandinavia in 2007–2009, there are no indications that wild or semi-domesticated reindeer were exposed to BTV. Recent serological screenings in Norway and Finland revealed no antibodies against BTV in semi-domesticated reindeer (30). Prophylactic immunization of susceptible populations seems to be the most effective way of controlling the disease, but after the declaration of Germany as a Bluetongue disease-free country in 2012, the vaccination against BTV was forbidden and it is therefore not available for the captive reindeer population and other captive wild ruminants (58). Four seropositive adult reindeer (6.0%) were detected in our study, indicating that reindeer were exposed to BTV, but there are no indications these animals became sick upon exposure. As samples were taken in 2013, the fact that only adult animals showed antibody titers fits nicely to the elimination of the circulation of the virus in Germany 2 years earlier.

Seven reindeer had antibodies against MCFV-related gammaherpesvirus (5.9%), which is in line with the prevalence detected in wild and semi-domesticated reindeer in Fennoscandia (17, 18) and caribou in Alaska (59). Transmission of MCFVs to reindeer in zoo settings may be associated with the contact of the animals with captive wild sheep (*Ovis* spp.) and goat species (*Capra* spp.) (15). Due to the low prevalence of antibodies against these pathogens, it was not possible to study this relation in our MCA model. With the lack of a suitable vaccine against MCFVs in reindeer, it would be recommended not to keep them in close proximity to sheep and goat species (15).

An eradication campaign against BVDV (Pestivirus A and B) has been enforced in Germany since 2011 and it is considered to be in the final stage of eradication (60). However, screenings for BVDV antibodies in wild ruminants in Europe reported the incidental spillover of the virus from cattle to cervids (28, 61). The detection of antibodies against pestivirus in reindeer in this study (4.2%) indicates the exposure to a virus from this genus in the captive reindeer in Germany. However, antibodies against pestivirus are routinely detected in semi-domesticated reindeer from BVDV-free countries, i.e., Norway, Sweden and Finland (18, 23, 24). These findings, together with the isolation of a pestivirus (V60-Krefeld; Reindeer-1), phylogenetically and antigenically more closely related to Pestivirus D (former BDV) than Pestivirus A or B (former BVDV1 and BVDV2) in Duisburg Zoo (Germany) (21, 22), suggest that the pestivirus in question may not be Pestivirus A or B, but rather a virus more specific to reindeer or cervids. However, further studies characterizing the pestivirus infecting zoo-kept reindeer are necessary to draw any firm conclusions about the nature of the exposure.

Serological screenings have demonstrated the presence of antibodies against SBV in a variety of wild ruminants in Europe (28). However, serological screening of 187 wild (2010–2013) and 450 semi-domesticated reindeer (2013–2015) in Norway, and 635 semi-domesticated reindeer in Finland, all *R. t. tarandus*, revealed no antibodies against SBV (30) and, to our knowledge, there are no reports on SBV in reindeer. A seroprevalence of 59.3% was detected for SBV in this study (Table 3). Adult (75.8%) and juvenile animals (58.3%) had a significantly higher seroprevalence than calves (14.3%), suggesting that most animals could have been exposed during the 2011–2012 outbreaks in Germany. The seroprevalence in our study was comparable to the one in German cattle (61.0%) and Belgian roe deer (63.0%) in the same period (27, 61). The lower seroprevalence detected among calves is in line with the fact that SBV was only sporadically detected in 2013 (62), when the outbreak was fading out.

Anti-*Brucella* antibodies were detected in one healthy reindeer. The ELISA used in this study detects antibodies against smooth *Brucella* spp. lipopolysaccharides (LPS) in reindeer (46). *B. suis* biovar 4 is the only *Brucella* species isolated from *Rangifer* (32), but to our knowledge, *B. suis* biovar 2 is the only one known to occur in Germany thus far, with seroprevalence rates up to 28.5% in wild boar depending on the geographic region in the country (63). *Brucella suis* biovar 2 has been reported to infect cattle, and spill over from wild boar was assumed to be the source of infection (64).

Serological cross-reactions and false positives may occur when detecting anti-*Brucella* antibodies. In cattle, an immune response of the animal to other microorganisms sharing epitopes with *brucellae* O-polysaccharides (65), like e.g., *Yersinia enterocolitica* O:9, may cause false positives (66). Investigation of fecal samples (*n* = 2,243) from eight herds of semi-domesticated reindeer in Norway and Finland yielded detection of *Yersinia* spp., but no detection of *Y. enterocolitica* O:9 (67). Since also other microorganisms may be serologically cross-reacting agents, serological results should always be interpreted with caution and the gold standard in brucellosis diagnostics still remains bacterial isolation.

One of the most common health challenges for captive reindeer are parasitic diseases (3). For *T. gondii*, large differences in seroprevalence have been reported among reindeer and caribou populations, from 0.9 to 1.0% in wild and semi-domesticated reindeer in Fennoscandia (38, 68) to 37.0% in barren-ground caribou (*R. tarandus groenlandicus*) in Canada (69). *Toxoplasma gondii* antibodies were detected in 52.1% of the studied zoo reindeer population, with 76.1% of the adult reindeer being exposed to the parasite. In this study, MCA revealed a positive relation between the presence of antibodies against *T. gondii* and the presence of vegetation, while it was negatively associated with the presence of carnivores in neighboring enclosures. These findings may be explained by the ecology of toxoplasmosis, with domestic and wild cats being main hosts and their feces being the carriers of the infective oocysts. The presence of vegetation may increase the presence of small rodents, common cat preys, in the reindeer facilities, while the absence of other predators in the vicinity may also contribute to the colonization of the area by domestic and feral cats which can contaminate the pasture and other food resources with their feces (35). With this information in mind, pasture maintenance, together with rodent and cat control would probably help to reduce the prevalence of toxoplasmosis in captive reindeer. However, a review paper by Hide et al. (70) discussed the vertical transmission of *T. gondii* as an important factor in the ecology of this parasite in sheep, with congenital transmission in up to 66% of pregnancies. Nevertheless, the importance of vertical transmission in sheep and other animals is still under discussion (70), and further studies in reindeer should be conducted in order to clarify if that is also the case in this species.

*Anaplasma phagocytophilum* is known to cause tick-borne fever in cattle and infect free-ranging wild ruminant species in Germany (40, 71, 72). The finding of *A. phagocytophilum* DNA in reindeer blood and in ticks from the same animals confirms the role of ticks as vectors, also in zoo-kept animals. However, the lack of clinical signs of disease in the studied population suggest that subclinical anaplasmosis may be more common than clinical infections in captive reindeer. No specific risk factors could be identified by MCA. The genetic types 16S-22Y, 16S-21X, and 16S-8J are well-known to be present in a variety of wild cervids in Europe, but, although sometimes detected in cervids, 16S-20W is mostly found in cattle (72). This further provides evidence for the interspecies exchange of this pathogen in particular in the context of a zoo. Male ticks which rarely feed on hosts showed almost the same prevalence rate as females. Transstadial transmission for *A. phagocytophilum* has been reported in ticks and may explain the high prevalence in males (73). However, since infected animals remain life-long carriers, subclinical infections could have been maintained without the regular presence of ticks in the enclosure (39). Anaplasmosis should definitely be included in the differential diagnosis whenever zoo-kept reindeer show signs consistent with tick-borne fever.

Most animal facilities have routines for addressing health parameters and infectious diseases, also in animals not displaying clinical signs, and especially for import and export purposes. However, local vector populations, such as ticks, but maybe also mosquitos and midges, should be screened for pathogens like BTV, SBV, *A. phagocytophilum* and others.

## Conclusions

The results of our analyses confirmed the exposure to all tested pathogens in the selected captive reindeer populations in Germany. The captive reindeer populations may thus serve as reservoir hosts for important pathogens that are circulating in local domestic, captive, and wild ruminant populations and arthropod vectors. These findings indicate that zoo animals should be included in national surveillance and control programs. The detection of antibodies against BTV was of special interest, since this pathogen was included in the German surveillance programs at the time of the sampling. Furthermore, animals infected with BTV were detected in areas where the diseases were not reported in other species at the time of the sampling.

## Data Availability Statement

The datasets generated for this study can be found in https://dataverse.no/dataset.xhtml?persistentId=doi:10.18710/4PQKKQ.

## Ethics Statement

Blood samples were taken for preventive medical care and leftovers were used for this study. When this was not the case, a research proposal was submitted to the appropriate authorities and approved (Landesamt für Landwirtschaft, Lebensmittelsicherheit und Fischerei Mecklenburg-Vorpommern, file number 7221.3-2-034/13). All procedures were performed in compliance with relevant laws.

## Author Contributions

JS and LG organized the dataset. JS, LG, and AO wrote the first draft of the manuscript. MT and MP secured funding for the study and organized the sampling and analyses. AO conducted the statistical analyses. FA-M conducted the multivariate analyses. LG did the sampling of animals. AO, NK, and MP contributed to the processing and examination of the samples. All authors contributed to the laboratory analyses and contributed to the writing and accepted the final version of the manuscript.

### Conflict of Interest

LG was employed by Zoo Duisburg AG. The remaining authors declare that the research was conducted in the absence of any commercial or financial relationships that could be construed as a potential conflict of interest.
