# Supplementary material for: A Multi-Pathogen Screening of Captive Reindeer (Rangifer tarandus) in Germany Based on Serological and Molecular Assays
Source: Front Vet Sci. 2019 Dec 20;6:461. doi: 10.3389/fvets.2019.00461 (PMC6933772; doi:10.3389/fvets.2019.00461)
Supplement: Supplementary file 1 [file Data_Sheet_1.pdf]

APPENDIX 1 | Individual plots of the multivariate analyses for presence of antibodies against alphaherpesvirus, bluetongue virus, MCF-related gammaherpesvirus, pestivirus, Schmallenberg virus, *Brucella* spp., *Neospora caninum* and *Toxoplasma gondii*, and the presence of *Anaplasma phagocytophilum* DNA in the analyzed reindeer. Black dots represent negative cases, gray dots represent positive cases and blue triangles represent the explanatory variables.

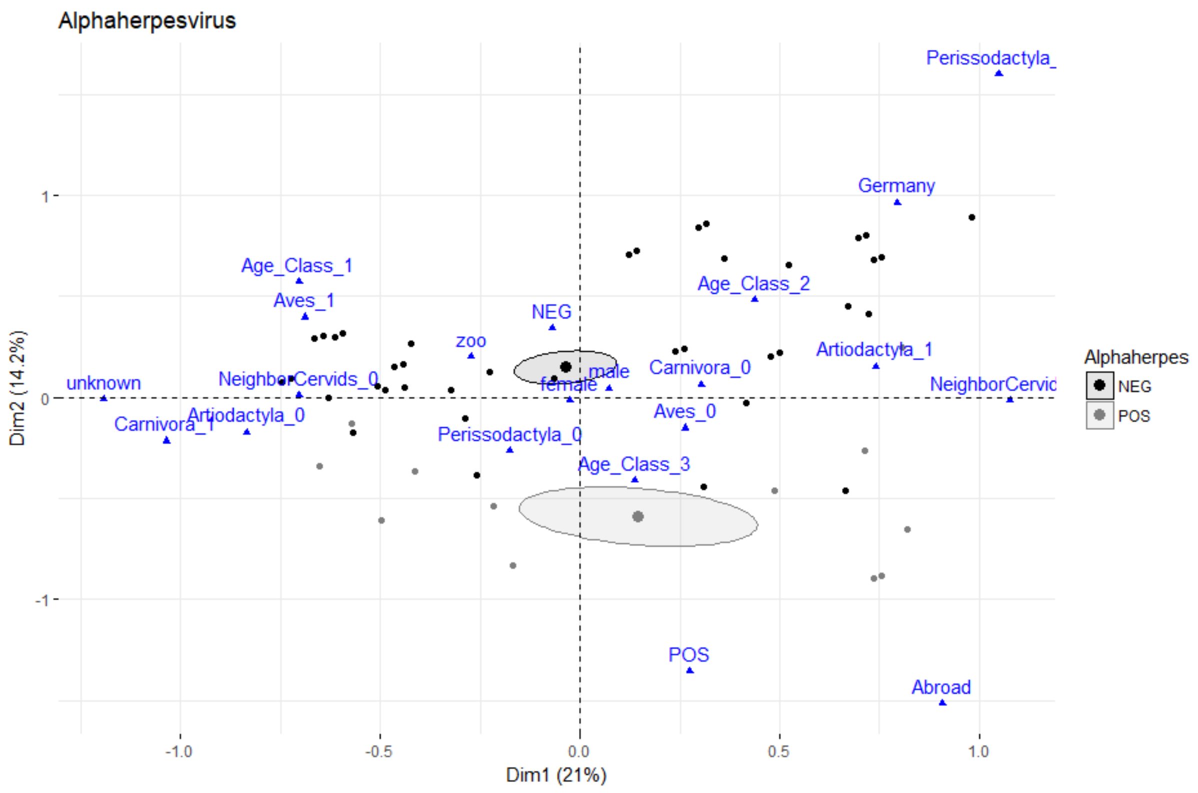

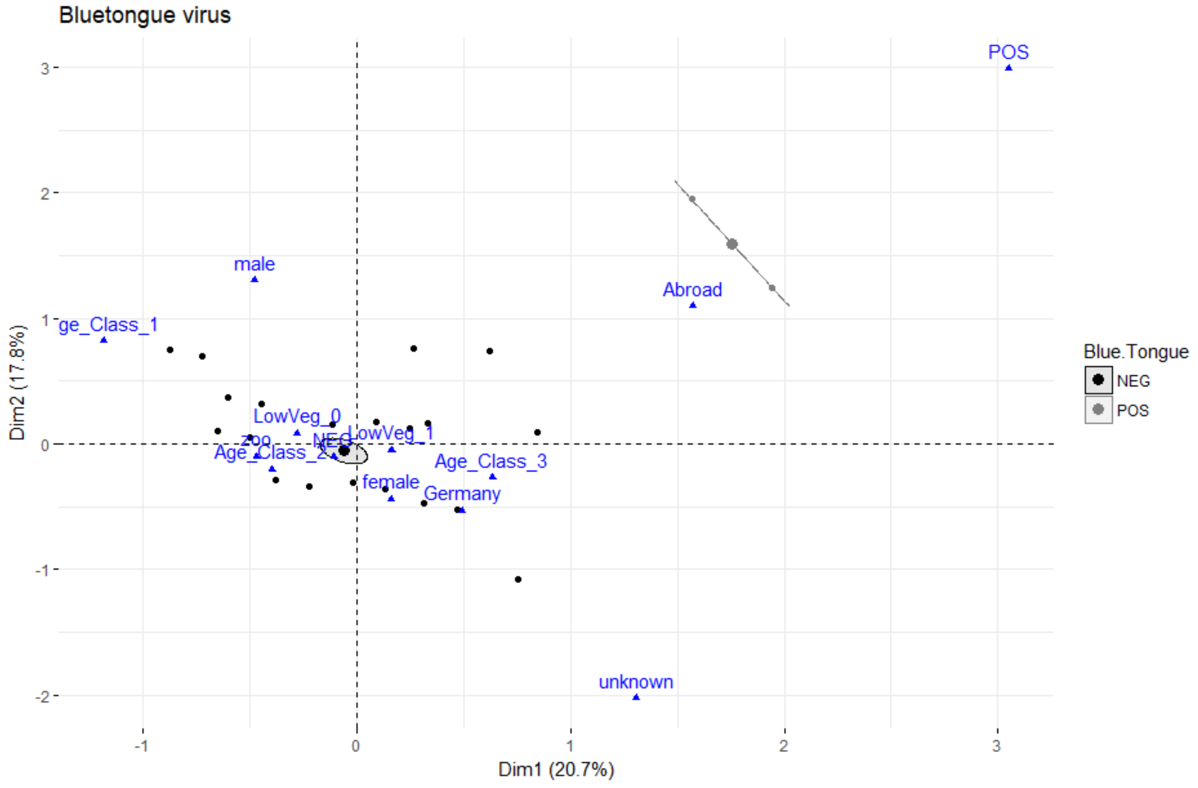

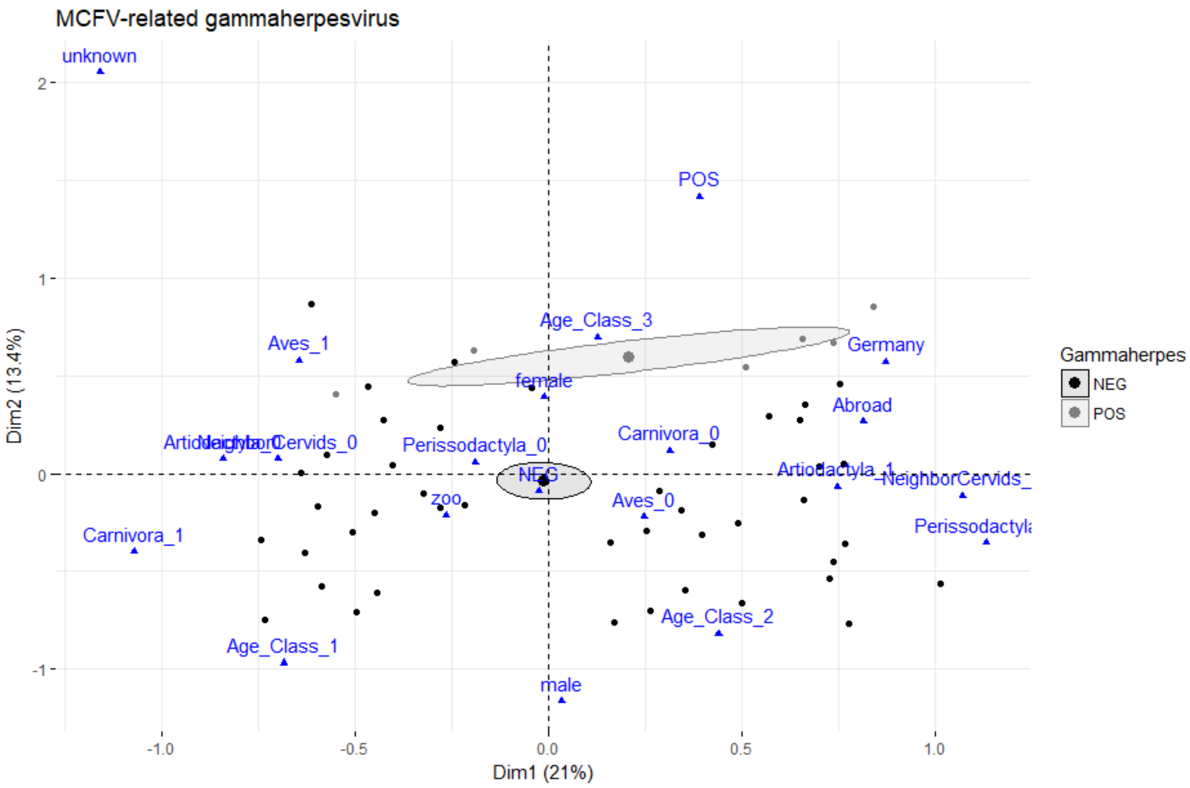
MCFV-related gammaherpesvirus
unknown
POS
Age_Class_3
Aves_1
Germany
female
Abroad
Perissodactyla_0
Carnivora_0
NEG
Artiodactyla_1
NeighborCervids_
zoo
Aves_0
Carnivora_1
Age_Class_2
Age_Class_1
male
Gammaherpes
NEG
POS
Dim1 (21%)
Dim2 (13.4%)
2
1
0
-1
-1.0
-0.5
0.0
0.5
1.0

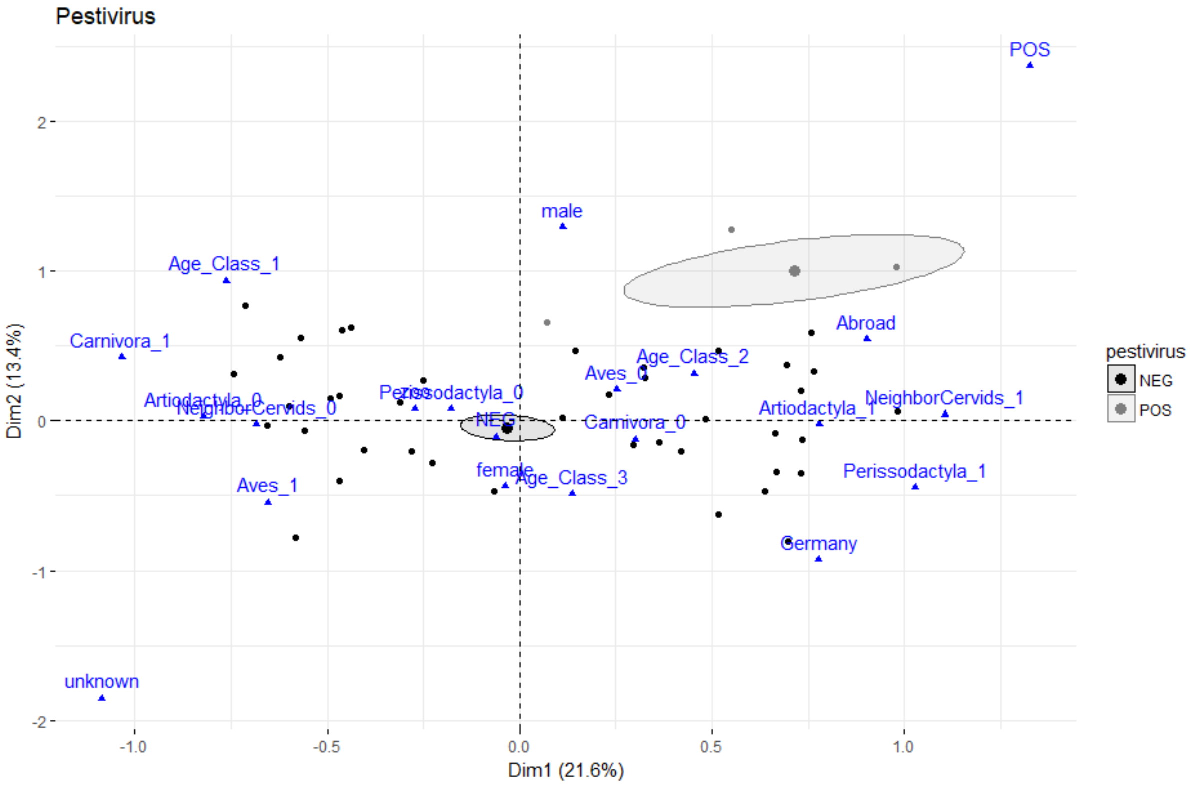
Pestivirus
POS
male
Age_Class_1
Abroad
Carnivora_1
Age_Class_2
Aves_0
Perissodactyla_0
Artiodactyla_1
NeighborCervids_1
NEG
Carnivora_0
Aves_1
Age_Class_3
Perissodactyla_1
Germany
unknown
pestivirus
NEG
POS
Dim1 (21.6%)
Dim2 (13.4%)
2
1
0
-1
-2
-1.0
-0.5
0.0
0.5
1.0

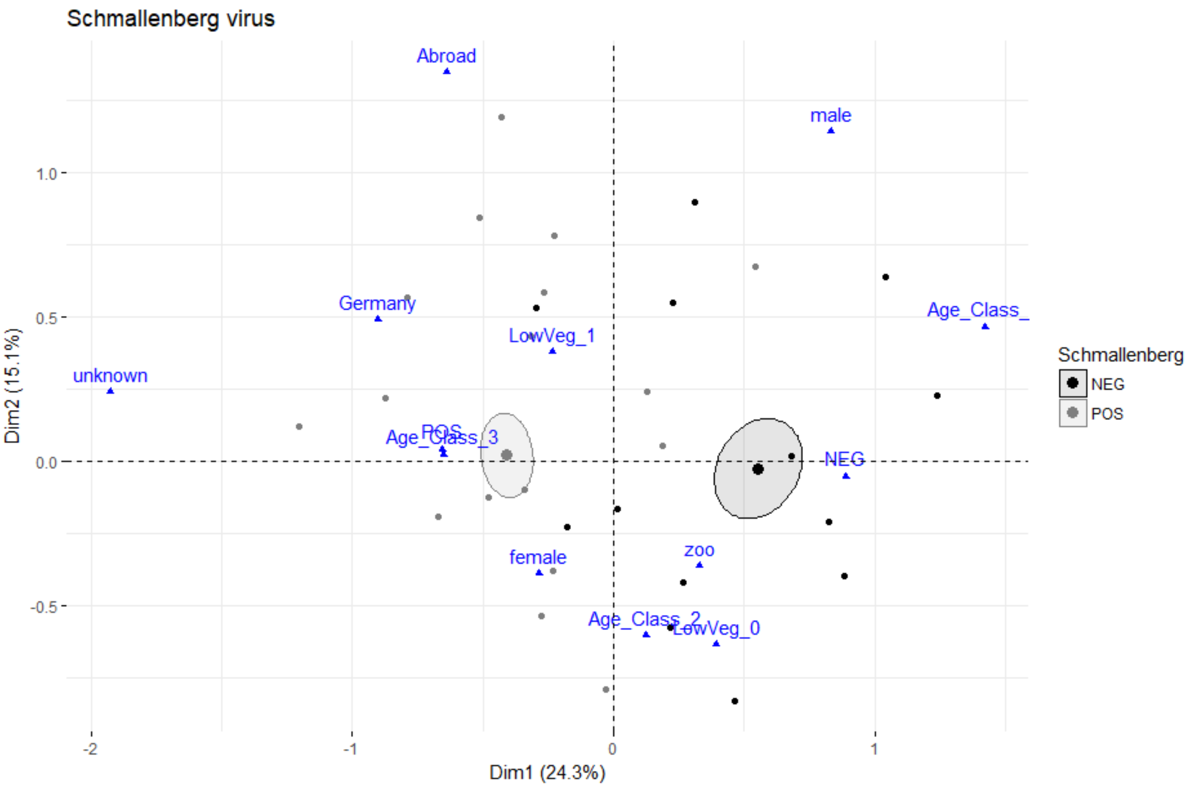
Schmallenberg virus
Abroad
male
Germany
LowVeg_1
Age_Class_
unknown
POS
Age_Class_3
NEG
female
zoo
Age_Class_2
LowVeg_0
Schmallenberg
NEG
POS
Dim2 (15.1%)
Dim1 (24.3%)
1.0
0.5
0.0
-0.5
-2
-1
0
1

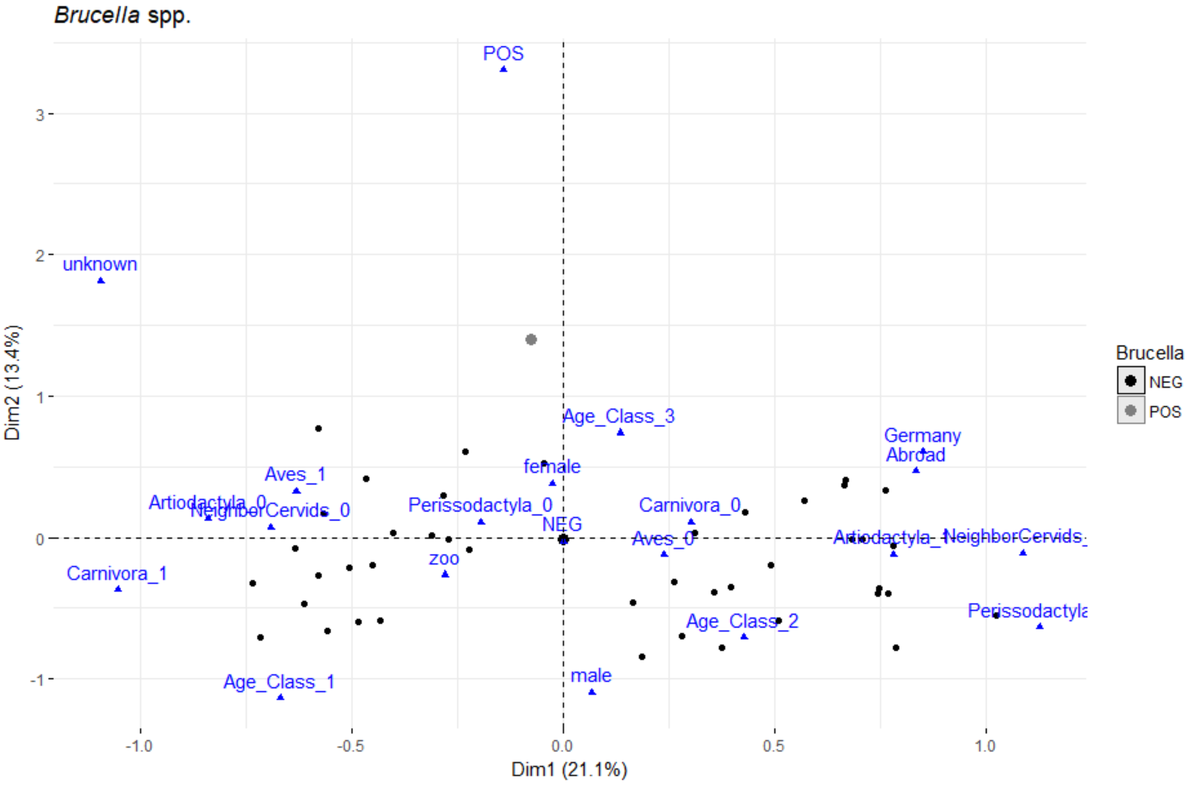
Brucella spp.
POS
unknown
Age_Class_3
Germany
Abroad
female
Aves_1
Artiodactyla_0
NeighborCervids_0
Perissodactyla_0
Carnivora_0
NEG
Aves_0
zoo
Carnivora_1
Age_Class_2
Perissodactyla
Age_Class_1
male
Brucella
NEG
POS
Dim2 (13.4%)
Dim1 (21.1%)
3
2
1
0
-1
-1.0
-0.5
0.0
0.5
1.0

Neospora caninum

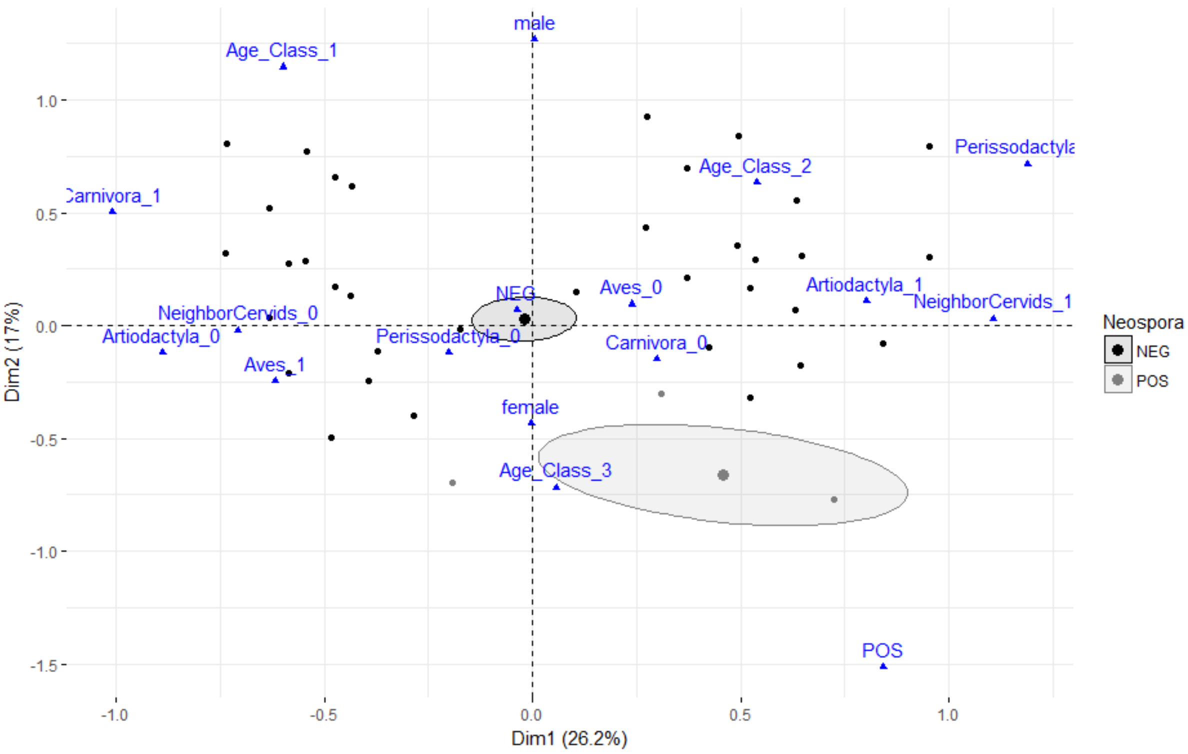

Toxoplasma gondii

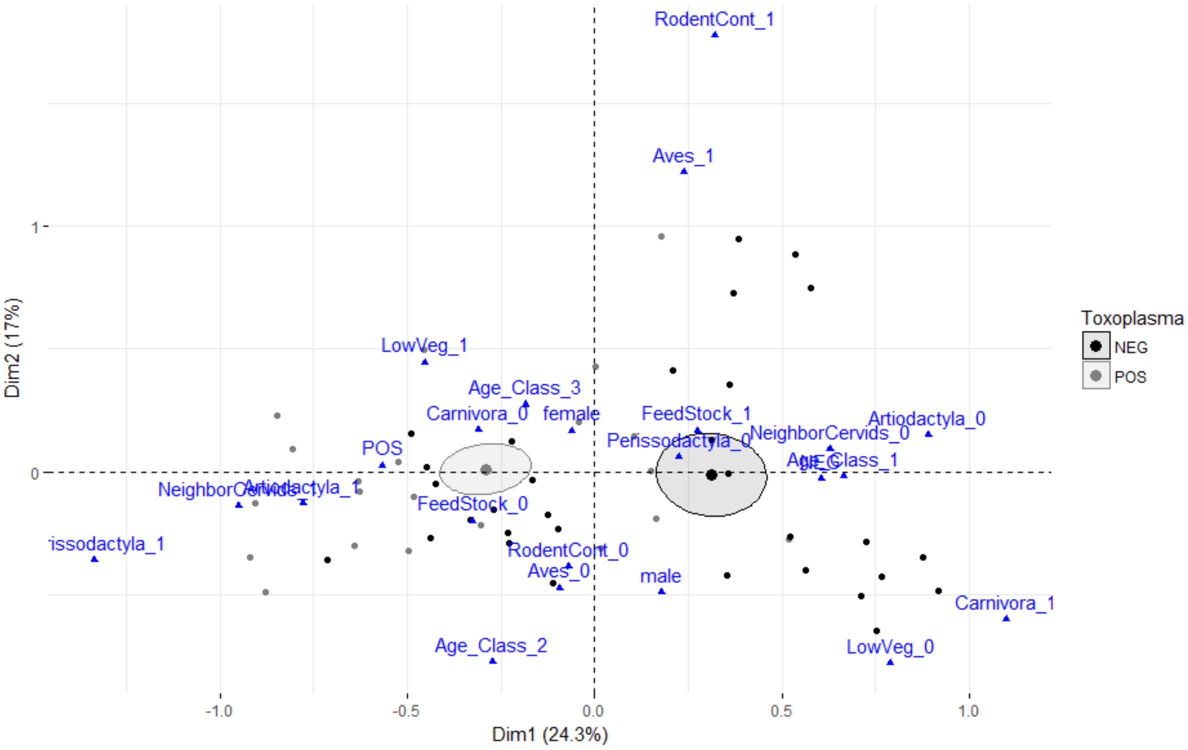

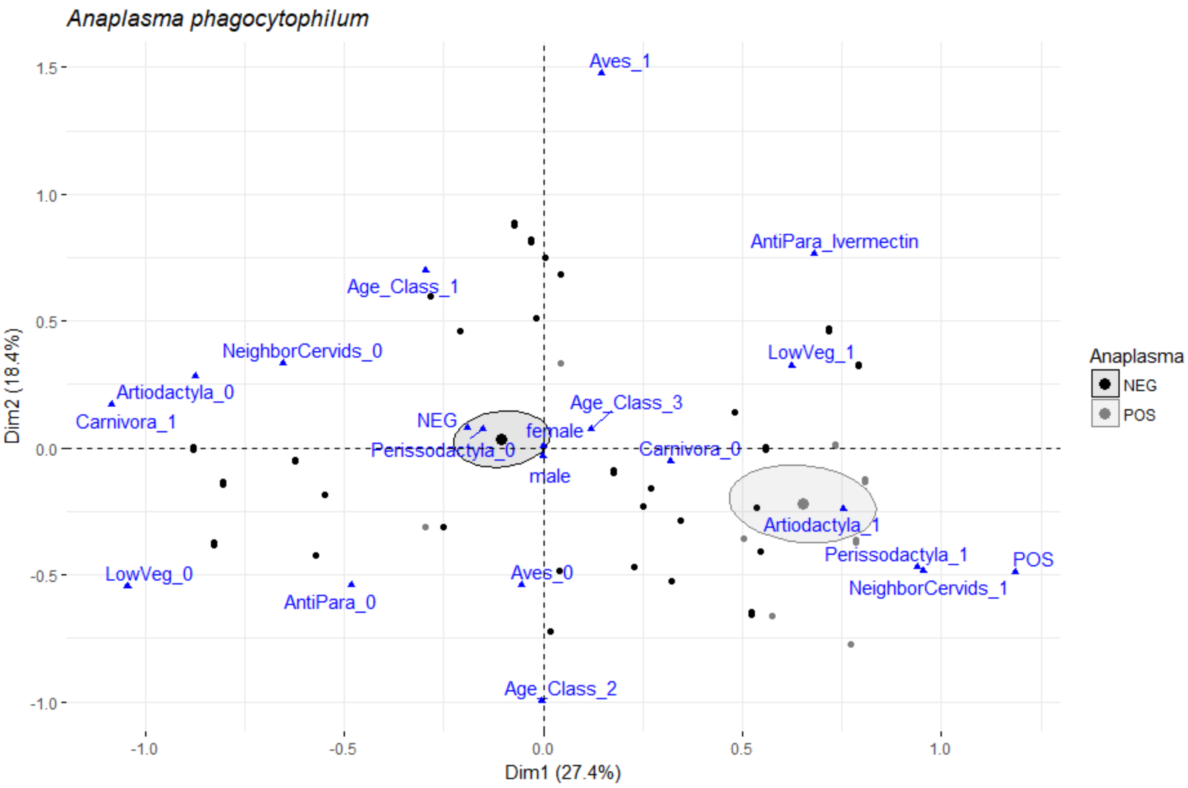

Anaplasma phagocytophilum
Aves_1
AntiPara_Ivermectin
Age_Class_1
NeighborCervids_0
LowVeg_1
Artiodactyla_0
Carnivora_1
Age_Class_3
NEG
female
Perissodactyla_0
Carnivora_0
male
Artiodactyla_1
Perissodactyla_1
POS
LowVeg_0
Aves_0
NeighborCervids_1
AntiPara_0
Age_Class_2
Dim1 (27.4%)
Dim2 (18.4%)
Anaplasma
NEG
POS
